# Conservative management of CIN2 p16 positive lesions in women with multiple HPV infection

**DOI:** 10.1186/s12879-020-05530-5

**Published:** 2020-10-29

**Authors:** Maria Teresa Bruno, Guido Scalia, Nazario Cassaro, Maria Costanzo, Sara Boemi

**Affiliations:** 1 Department of General Surgery and Medical Surgery Specialties, Gynecological Clinic, University of Catania, Catania, Italy; 2 Department of Biomedical and Biotechnological Sciences, Clinical Virology, University of Catania, Catania, Italy; 3 Gynecological Oncology, Humanitas, Catania, Italy

**Keywords:** HPV infection, Multiple HPV infection, CIN2, p16 protein, Colposcopy, Laser-therapy, LEEP

## Abstract

**Background:**

According to the 2006 American Society for Colposcopy and Cervical Pathology guidelines, positive CIN2 p16 in women over the age of 25 should be managed with excisional treatment. However, excisional treatment is associated with physical, psychological and obstetric morbidity and can have a negative impact on sexual function. In our study we sought to identify a clear management strategy, addressing the impact of routine use of p16 immunohistochemistry in this population and identify appropriate criteria for patient selection with the aim of reducing over-treatment.

**Method:**

We studied the medical records of 130 patients who had undergone laser therapy for CIN2. Each patient underwent colposcopy, biopsy and HPV test and were tested for p16 protein,. Patients were divided based on HPV infection into: single infections, multiple infections. All patients underwent ZTA laser therapy with follow-up (2-year follow-up).

**Statistical analysis:**

Contingency tables were created to evaluate the correlation between single, multiple and CIN2+ infections. Values with *p* < 0.05 were considered statistically significant.

**Results:**

Single infections had a histological regression of 61.8% (21/34) and a histological persistence rate of 35.3% (12/34), which was greater than the multiple infection rate. The common characteristic that the women with persistence and progression had was the dimension of the lesion and the genotype 16. Ten cases of histological persistence and the only case of progression had one lesion greater than three quarters of the cervix.

**Conclusions:**

With the progress of our understanding of the natural history of infection from human papillomavirus and the increasing use of colposcopy, thanks to the addition of HPV genotyping and the technique of immunohistochemistry, conservative management of these lesions is now possible.

## Background

Cervical cancer is still a major cause of cancer death in women around the world [1, 2], particularly in areas with scarce resources. Human papillomavirus (HPV) infection is a sexually transmitted disease whose prevalence of various genotypes shows significant differences worldwide [3]; to date, over 100 types of HPV have been identified, 18 of which are associated with cervical carcinogenesis. Persistent infection with High-Risk Human Papilloma Virus (hr HPV) is necessary for the development, maintenance and progression of CIN (Cervical Intraepithelial Neoplasia) lesions. However, only a small percentage of infected women develop CIN2 + (CIN2, CIN3, SCC). The diagnosis of CIN is notoriously complicated due to the variability of interpretation of the clinical picture among anatomical-pathologists [4]. In particular, while CIN1 and CIN 3 are easily diagnosed and cause no doubts for pathologists, CIN2 is an equivocal diagnosis, poorly reproducible, which includes lesions that behave like CIN1 and CIN3 so much so that many experts doubt the existence of CIN2. Women under 25 years of age eliminate HPV efficiently, registering a spontaneous regression of CIN within two years; 70–94% of low-grade squamous intraepithelial lesions (LSIL); 65% of high-grade squamous intraepithelial lesions (HSIL) [5], leading to recommendations to not immediately treat CIN2 in young women [6]. The current knowledge on CIN2 regression rates in women over 25 years of age is poor [5]. Uncertainty regarding the natural history of CIN2 also casts doubt on its potential for spontaneous regression, and it is possible that the regressive nature of CIN2 depends, in some way, on the single pathologist who carried out the diagnosis.

In March 2012, the College of American Pathologists and the American Society for Colposcopy and Cervical Pathology in collaboration with 35 stakeholder organizations, held a consensus conference called the LAST Project (Lower Anogenital Squamous Terminology). The recommendations of this project include the use of a uniform two-level terminology, in parallel with the terminology of the cytological relationship of the Bethesda System, to describe the histology of the squamous disease associated with human papillomavirus in all the tissues of the anogenital tract. The two levels reflect the biology of the viral infection: one infective or productive phase, low-grade squamous intraepithelial lesion (LSIL), and a true preneoplastic lesion, high-grade squamous intraepithelial lesion (HSIL). The recommendations of the LAST Project offer a solution for the equivocal category of CIN2, which cannot be differentiated in a reliable way only from histopathological data.

The LAST Project (Darragh, 2012) [7] shows that the addition of p16 staining significantly improves the reliability of CIN2 diagnosis, [8] the use of p16 is advised to confirm the diagnosis of a high-grade lesion when there is a diagnosis of CIN2 based on hematoxylin and eosin morphology. If a sample of “CIN 2” is positive for p16, it should be classified as “HSIL”; if p16 is negative, it should be classified as “LSIL”. Positive p16 staining of squamous cells in all the thickness of the epithelium correlates well with the diagnosis of HSIL. p16 is already widely used by pathologists in addition to cervical histopathology. From here we have the therapeutic implications that help the clinician: CIN2 p16 negative is equal to CIN1, undergoes follow-up, CIN2 p16 positive (promoted to CIN3) undergoes excisional treatment according to the guide lines of the American Society for Colposcopy and Cervical Pathology of 2006, with the aim of identifying misdiagnosed invasive or micro-invasive forms on the histological sample (found in up to 8–12% of the cones carried out for CIN2–3/CIS). These treatments are destructive and can be associated with acute side effects and long-term reproductive morbidity. Are we sure that the excisional treatment of CIN2 p16 positive is not an overtreatment?

The aim of this study was to evaluate the efficacy of conservative treatment of high-grade intraepithelial cervical neoplasia (CIN2 p16 positive) by means of CO^2^laser by means of the evaluation of relapse at the 2-year follow-up.

## Methods

We studied the medical records of 130 patients who, from April 2015 to April 2017, had undergone laser therapy for CIN2 at the outpatients’ clinic of Colposcopy of the Gynecology Unit of the University Hospital of Catania (University of Catania, Italy) and we selected the women who met the following inclusion criteria:

- Women between the ages of 25 and 45;
- Patients with histological diagnosis of CIN2;
- Protein p16 positive;
- Patients who had undergone HPV test;
- Patients with visible squamo-columnar junction;
- Women without cervical pathology previously;
- Patients without immune system pathologies;
- Patients who had completed at least two years of follow-up;
- Patients who were not pregnant.

Only 64 patients met the inclusion criteria, from whom we obtained the following clinical data: patient’s age, type of pathology, HPV sample, lesion dimensions, method of treatment, data of follow-ups, pretreatment viral genotype, post-treatment HPV genotype and cases of relapse. Each patient underwent colposcopy, specific biopsy and an HPV test. Patients were divided based on HPV infection into: single infections (34 cases), multiple infections (30 cases). All patients were tested for histologic p16 protein, and then underwent ZTA laser therapy with follow-up. The follow-up was carried out every six months for the first year after treatment then once a year.

At each follow-up the patients were examined by Pap test and colposcopy, moreover, cytological ecto-endocervical samples were taken and placed in a ThinPrep solution. The samples were then sent to the laboratory for total DNA and RNA extraction and for viral DNA genotyping by means of genetic amplification followed by hybridization with specific probes for genotypes able to identify most HPV genotypes of the genital region [28 genotypes of high-risk HPV (16, 18, 26, 31, 33, 35, 39, 45, 51, 52, 53, 56, 58, 59, 66, 68, 73, 82), low-risk genotypes (6, 11, 40, 43, 44, 54, 70) and undefined risk (69,71,74)]. The commercial method used was the MAG NucliSenseasy system (bioMerieux SA, Marct l’Etoile, France).

Colposcopy was carried out using a Zeiss OPM1F colposcope (Carl Zeiss, Jena, Germany) and applying acetic acid and a solution of Lugol’s iodine.

The HPV genotype was classified as follows: 1) negative; 2) HPV 16; and 3) hr. HPV (HPV18, 31, 33, 45 and other hr). The laser procedure was carried out in out-patient day-surgery by expert personnel. Vaporization was carried out using a Sharplan CO^2^ laser (ESC Sharplan, Yokneam, Israel) with a maximum power of 30 W, used continuously. The diameter of the beam varied from 0.5 to 1 mm, guided by a micromanipulator, to an average depth of vaporization of 6 mm. Power varied from 600 to 1200 W/cm ^2^. The CO^2^ laser was connected to a Zeiss OPMI colposcope (Carl Zeiss. Oberkochen, Germany). The distal limits of the lesion were delineated by 3% acetic acid. The final state after two years of follow-up was classified as progression if the histologic showed a CIN3, persistence if the lesion was CIN2, regression if the histologic was negative or LSIL/CIN1, viral persistence if the HPV test was positive for the same genotype as after treatment.

### Statistical analysis

The statistical analysis of the data was carried out with the software package SPSS 15.0 (SPSS Inc.; Chicago, IL, USA). The analysis of the data was made using the χ2 test and, if necessary, Fisher’s exact test was used to calculate the statistical significance (*p* value) of the difference between groups. Contingency tables were created to evaluate the correlation between single, multiple and CIN2+ infections. Values with *p* < 0.05 were considered statistically significant.

## Results

The average age of the 64 women included in the study was 36.4 years. Progression, regression and persistence rates of CIN2 are shown in Table 1. The tests used during follow-up in the study group are shown in Table 2. At the end of the 24 months of monitoring, we found a histological regression rate of 78.1% (50/64), a viral persistence rate of 50% (25/50) and 13 cases of histological persistence (20.3%). One case of progression to CIN3 was found at the 24-month follow-up. Among the women who had regression (50 cases), this was found at the six month follow-up in 36 cases. The prevalent genotype was HPV16 (41/64). Stratifying the women by type of HPV infection, single infections had a histological regression of 61,8% (21/34), the histological persistence rate, 35,3% (12/34), was greater than the multiple infection rate. We had 8 cases of viral persistence (33,3%) and one case of progression (Table 3). Multiple infections had 96.7% (29/30) histological regression, 58.6% viral persistence (17/29) and only one case of histological persistence, 3.3% (1/30), there were no cases of progression (Table 4). The single infection had an OR of 17.95 (CI 95% = 2.18–148.09) compared to multiple infection.

**Table 1:** Prevalence of histological regression, persistence or progression after an follow-up of 24 months in the study group

	Regression		Persistence	Progression	Total
	histological regression	viral persistence	histological and viral persistence		
**Total**	50/64 (78.1%)	25/50 (25%)	13/64 (20.3%)	1/64 (1.6%)	64
**Single infection**	21/34 (61.8%)	7/21 (33.3%)	12/34 (20.3%)	1/34 (2.9%)	34
**Multiple infections**	29/30 (96.6%)	17/30 (58.5%)	1/30 (3.4%)	0	30

**Table 2:** Tests used during follow-up in the study group

0utcome	Total	HPV Test		Histology	ColposcopyExame
		pos	neg		
Progression	1	1	0	1 CIN3	Type1 TZ
Persistence	13	13	0	13 CIN2	Type1 TZ
Regression	50	25	25	50 CIN1/ neg	Type1 TZ

**Table 3:** Prevalence of histological regression, persistence and progression for HPV genotypes in the study group with a single infection

Genotype	Regression		Persistence	Progression	Total
	histological regression	viral persistence	histological and viral persistence		
	21/34 (61,8%)	7/21 (33,3%)	12/34 (35,3%)	1/34 (2,9%)	34
HPV16	13	4	11	1	25
HPV31	3	1	0	0	3
HPV18	2	0	0	0	2
HPV51	2	1	1	0	3
HPV52	1	1	0	0	1

**Table 4:** Prevalence of histological regression, persistence and progression for HPV genotypes in the study

Genotype	Regression		Persistence	Progression	total
	histological regression	viral persistence	histological and viral persistence		
	29 (96.6%)	17 (58.6%)	1 (3.3%)	0	30
HPV 16	15 (51.8%)	8 (53.3%)	1 (100%)	0	16
others	14 (48.3%)	9 (64.3%)	0	0	14

group with multiple HPV infections (after a 24 months of follow-up)

As regards genotyping, the lesions were all caused by a high-risk genotype, even in multiple infection the most common genotype was 16 (16/30) 53.3%.

The common characteristic that the women with persistence and progression had was the dimension of the lesion on the cervix and genotype 16. Ten cases of histological persistence and the only case of progression had one lesion greater than three quarters of the cervix. Women with a colposcopic lesion greater than 3 quarters of the cervix had an OR of 16.67 (CI 95% = 2.27–122.22). The case of progression was treated with LEEP. All cases with viral persistence were invited to undergo HPV vaccine (data not available). There were no intra- or post-operatory complications with the laser treatment to an average depth of vaporization of 6 mm.

## Discussion

Spontaneous regression rates of CIN2 of women over the age of 25 vary between 40 and 74%. It is important to note that CIN2 is the least reproducible of all cervical diagnoses and it is possible that the “regression” of CIN2 is due to a subjective interpretation of the pathologist who made the diagnosis. Thus “equivocal” CIN2 lesions increase the overall regression rate and, as a consequence, the regression may have been reported in excess [9]. The diagnosis of CIN2 cannot be reliably differentiated only with histopathological data. The literature indicates a better agreement between pathologists for the diagnosis of CIN2 when the H&E morphology is used together with p16 compared to the H&E morphology alone. The p16 protein when positive in a CIN2 suspect gives us the confidence of having selected true CIN2 cases. These are the cases for which the spontaneous regression rate must be studied, therefore there is a need for a prospective study analysing the regression potential of true CIN2 (positive CIN2 p16) in women over the age of 25.

Not all preneoplastic lesions will progress to cancer, therefore regardless of the positivity of p16 we cannot yet accurately predict which lesions would become malignant if left untreated. According to the 2006 American Society for Colposcopy and Cervical Pathology guidelines [6], positive CIN2 p16 in women over the age of 25 should be managed with excisional treatments. Treatment should be effective in eradicating the CIN2 lesion and have minimum adverse effects on future fertility and pregnancy outcomes, particularly in young women. However, excisional treatments are associated with physical, psychological [10] and obstetric [11] morbidity and can have a negative impact on sexual function [12–14].

In our study we sought to identify a clear management strategy, address the impact of routine use of p16 immunohistochemistry in this population and identify appropriate criteria for patient selection with the aim of reducing over-treatment.

LEEP (Loop Electrosurgical Excision Procedure), as an excisional procedure of the cervix, is the most used surgical treatment for programs of cervical screening in developed countries [15]. Some advantages of LEEP have been shown, among which is the excision of the entire transformation zone, the conservation of tissue samples for histological evaluation, low-cost equipment and the rapid rehabilitation of the cervix [11]. Thanks to these advantages, both in diagnosis and in CIN treatment, the ASCCP recommends LEEP as the excisional procedure for CIN2 +. Kyrgiou et al. [11] reported higher rates of preterm membrane rupture after LEEP compared to ablative surgery, indicating that ablative surgery such as laser vaporization should be chosen as the least invasive surgery for CIN. In a meta-analysis [16], LEEP was associated with significantly higher risks of perinatal mortality and preterm birth. Cochrane’s revision reported three comparative studies on laser ablation with respect to LEEP [15] and did not show significative differences in the risk of residual disease in the women who had received laser ablation or LEEP. Other authors [17] reported a relapse of only 2.5% at 5 years after laser treatment of high-grade lesions, with adequate biopsies, with completely visible margins, carried out by colposcopy experts.

In the laser treated women in this study, we found only one case of progression to CIN3 and no relapse at 2 years of follow-up. Multiple infections showed a greater regression rate, unlike single infections with a greater rate of histological persistence. Undoubtedly, single infection, the presence of genotype 16 and one lesion greater than three quarters of the cervix are positively associated with persistence and progression, Castle [5, 18, 19] reported that CIN2 caused by HPV 16 may be more likely to progress than CIN2 caused by other HR genotypes. In our study, we noticed that cases associated with multiple infection respond well to conservative therapy. Of course, the selection of cases to be treated is vital and, given that the literature [20] shows a risk of recurrence for up to 10 years for ablative treatments, strict post-treatment follow-up protocols are necessary, which advise the use of the HPV test. In recent years, several studies have shown that the HPV test performed 6–12 months after treatment is more sensitive than cytology in identifying women with recurrent disease and has a very high negative predictive value [21–23].

Therefore, in patients correctly selected such as non-immunocompromised women and young women (younger than 25 years) with infections from HPV16 (CIN2 in the presence of HPV 16 has less probability of regression), cases in which the squamo-columnar junction is completely visible, the lesion is not very extended, and with multiple infections, a more conservative management of CIN2 p16 positive lesions is possible. Not with standing the limits of the retrospective analysis of the data, our results indicate that laser therapy is efficacious in the conservative treatment of CIN2 p16 positive lesions in carefully selected women. The persistence of the infection from high-risk HPV at follow-up is a significative predictive factor of residual or recurrent CIN after surgery [24, 25]. Vaccination with the nonovalent HPV vaccine [26] among the patients aged between 20- and 45-years old undergoing treatment for CIN2 is a valid addition in the prevention of recurrence.

## Conclusions

With the progress in our understanding of the natural history of infection from human papillomavirus and the increasing use of colposcopy, thanks to the addition of HPV genotyping and the technique of immunohistochemistry, conservative management of these lesions is now possible. As cervical treatment has been associated with obstetric morbidity in a successive pregnancy it is possible to stratify the risk of women with CIN2 p16 and guide the clinical management towards conservative management or cervical treatment.

Therapeutic options have evolved in the procedure of local ablation for intraepithelial lesions of the cervix.

## Data Availability

The datasets used and/or analyzed during the current study are available from the corresponding author on request.

## Abbreviations

HPV: Human Papillomavirus
CIN2: Cervical Intraepithelial Neoplasia 2
hr. HPV: high- risk HPV
LEEP: Loop Electrosurgical Excision Procedure
ATZ: Transformation Anomaly Zone
HSIL: High-grade squamous intraepithelial lesions
LSIL: Low-grade squamous intraepithelial lesions
SCJ: Squamo-columnar junction
LAST: Lower Anogenital Squamous Terminology
